# Stricter Adherence to Dietary Approaches to Stop Hypertension (DASH) and Its Association with Lower Blood Pressure, Visceral Fat, and Waist Circumference in University Students

**DOI:** 10.3390/nu12030740

**Published:** 2020-03-11

**Authors:** Silvia Navarro-Prado, Jacqueline Schmidt-RioValle, Miguel A. Montero-Alonso, Ángel Fernández-Aparicio, Emilio González-Jiménez

**Affiliations:** 1 Department of Nursing, Faculty of Health Sciences, University of Granada, 52071 Melilla, Spain; silnado@ugr.es; 2 Department of Nursing, Faculty of Health Sciences, University of Granada, 60, 18016 Granada, Spain; anfeapa@ugr.es (Á.F.-A.); emigoji@ugr.es (E.G.-J.); 3 Department of Statistics and O.I. Faculty of Medicine, University of Granada, 18016 Granada, Spain; mmontero@ugr.es

**Keywords:** blood pressure, eating habits, university students, young adults, Dietary Approaches to Stop Hypertension (DASH)

## Abstract

How diet affects blood pressure (BP) in young adults has not been studied in sufficient depth. For this purpose, we analyzed adherence to the Dietary Approaches to Stop Hypertension (DASH) dietary pattern and BP in Spanish university students. The sample population of our cross-sectional study consisted of 244 subjects (18–31 years old), who were in good health. Measurements were taken of their systolic and diastolic BP. A food frequency questionnaire and 72 h food record were used to assess their dietary intake in the previous year. The resulting DASH score was based on foodstuffs that were emphasized or minimized in the DASH diet. Analysis of covariance adjusted for potential confounding factors showed that the mean values for systolic BP, visceral fat rating, and waist circumference (WC) of the subjects in the upper third of the DASH score were significantly lower than those of the subjects in the lower third (for systolic BP: mean difference −4.36 mmHg, *p* = 0.004; for visceral fat rating: mean difference −0.4, *p* = 0.024; for waist circumference: mean difference −3.2, *p* = 0.019). Stricter adherence to the DASH dietary pattern led to a lower BP, visceral fat rating, and WC values in these university students. Nevertheless, further prospective studies are needed to confirm these results.

## 1. Introduction

Cardiovascular diseases (CVDs) are the leading cause of early death in countries throughout the world [1]. With a global prevalence of approximately 26.4%, high blood pressure (BP) in young adults is regarded as a major public health problem [2]. Spain is hardly an exception as national studies have reported a prehypertension prevalence of 24% among university students [3]. Although elevated BP at a young age does not usually cause CVD, the development of hypertension is associated with the early onset of left ventricular hypertrophy, carotid wall thickening, and retinopathy [2]. In addition, young adults with abnormal BP are more predisposed to be hypertensive during midlife (40–65 years old) [4], with hypertension being the leading cause of premature death [5]. However, epidemiological studies indicate that a healthy lifestyle based on healthy eating habits is associated with lower BP, lower abdominal and visceral fat deposits [6], and thus lower cardiovascular risk [7].

Studies in university populations reflect the pervasiveness of unhealthy behaviors and lifestyles [8,9], especially unhealthy eating patterns [10]. Therefore, the university stage is critical for the establishment of nutritional behaviors, which may eventually become entrenched habits in the same way that high BP may become a life-long condition [11]. Therefore, the promotion of healthy habits among university students may lead to important long-term health benefits [11].

Currently, the effect of diet on the BP of young adults is not well understood [12]. Studies on the connection between diet and BP often focus on analyses of individualized nutrient intake, which does not clarify the biological mechanisms involved in this relationship [13]. Of the hypothesis-oriented methods for the identification of dietary patterns, those related to Dietary Approaches to Stop Hypertension (DASH) have been the focus of considerable research [14].

Accordingly, an umbrella review of meta-analyses by Dinu et al. [15] concluded that the DASH diet improves BP. In this same line, a cross-sectional study of adults by Phillips et al. [16] found that strict adherence to DASH was linked to a lower systolic BP. Moreover, the results of a randomized controlled trial of prehypertensive patients showed a significant reduction of systolic BP in patients following the DASH diet [17]. Nevertheless, up until now, most studies on the association between the DASH diet and BP have targeted adolescent or adult populations. To the best of our knowledge, there have been no studies of this association in young university students. For this reason, our study focused on adherence to the DASH dietary pattern and BP, visceral fat rating, and waist circumference (WC) in a sample of healthy Spanish university students.

## 2. Method

### 2.1. Study Design and Subjects

This cross-sectional study was performed during the 2013–2014 academic year, and 244 of a total of 1188 university students participated in the study. The participants had a mean age of 22.4 ± 4.76 years. Their selection was the result of a random sampling of students at the university campus of Melilla, a Spanish city situated on the northwest African coastline, opposite the provinces of Granada and Almeria in Spain. Melilla is a modern western city, characterized by great cultural richness stemming from the centuries-long coexistence of different ethnic groups and cultures.

### 2.2. Data Collection

**Figure 1:**
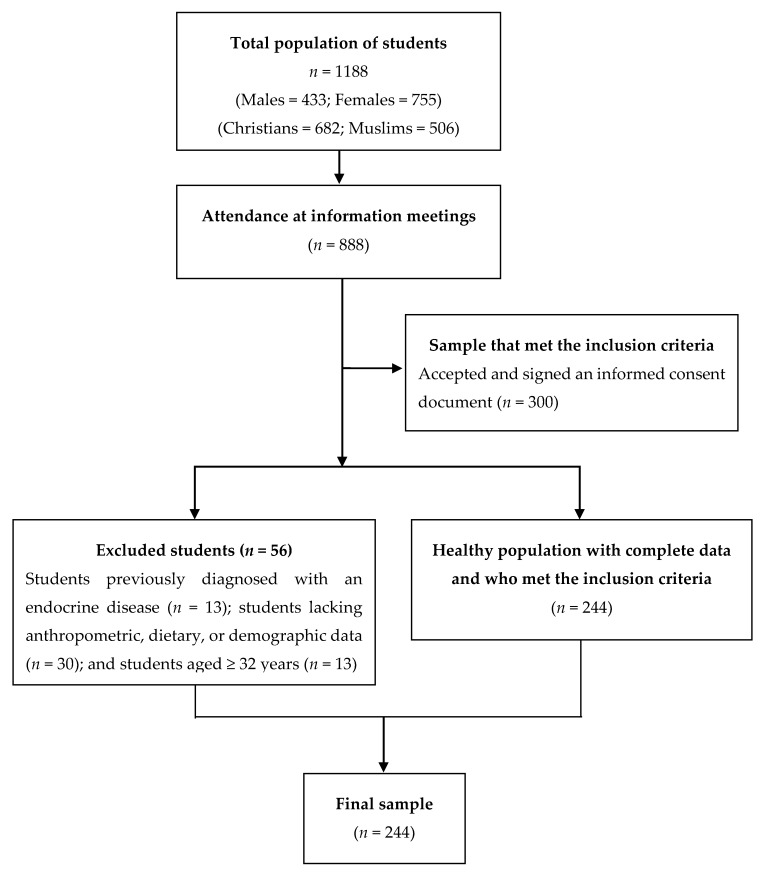
Flow diagram of the recruitment process.

The Melilla campus is composed of three university centers: (i) the Faculty of Education and Sport Sciences, (ii) the Faculty of Social Sciences and Law, and (iii) the Faculty of Health Sciences. To participate in the study, students had to be enrolled in a degree program offered by one of these three faculties. They were also required to give their informed consent. Students with a prior medical history of endocrine or metabolic diseases, as well as those who did not wish to sign the consent form, were excluded from the study. Figure 1 summarizes the recruitment process.

Information meetings were scheduled and held for all students (*n* = 1188) during September 2013 at the university campus of Melilla. Of the 1188 students, only 888 attended all meetings. At the meetings, participants learned about the different evaluations and questionnaires that they would have to complete to participate in the study. An informed consent form with a description of the study was given to all students attending the meetings. After applying the previously mentioned inclusion criteria, 300 students were selected for the study. However, 56 were subsequently excluded because of one of the following reasons: (i) previous diagnosis of an endocrine pathology (*n* = 13); (ii) incomplete anthropometric, dietary, or demographic data (*n* = 30); (iii) age ≥32 years (*n* = 13).

Accordingly, 244 students who complied with all of the inclusion criteria were selected as participants. In October 2013, each participant was given an anthropometric evaluation. Their body composition was also analyzed, and their dietary habits were assessed.

The study received the approval of the Ministry of Education and Youth of the Government of Melilla. Furthermore, the Ethics Committee of the University of Granada (Code 841) also approved the study as well as the informed consent form. All of the participants signed the informed consent document, and the confidentiality of their personal information was guaranteed by coding the data. This research was carried out in strict compliance with the international code of medical ethics established by the World Medical Association and the Declaration of Helsinki.

### 2.3. Blood Pressure

The BP of the participants was measured with a previously calibrated aneroid sphygmomanometer and a Littmann^®^ stethoscope. In this regard, the study followed the recommendations for BP measurement of the Subcommittee of Professional and Public Education of the American Heart Association Council on High Blood Pressure Research [18]. Each participant was requested not to eat, drink alcohol or caffeine, smoke, exercise, and bathe for at least 30 min before his/her BP measurement. During the BP measurement, the subject was seated on a chair for 5 min with his/her back supported, feet flat on the floor, and wrist relaxed at heart level. The results were interpreted according to the Korotkoff sounds: phase I, systolic BP; phase V, diastolic BP [18]. Coefficient of variation (CV%) of systolic BP was of 10.03%, while diastolic BP had a CV% of 13.89%.

### 2.4. Dietary Intake

Each participant filled out a comprehensive food frequency questionnaire (FFQ) consisting of 168 food items in order to assess his/her typical dietary intake during the previous year [19]. The objective was for them to record the frequency of consumption of each food item per day, week, and month.

Furthermore, a 72 h food record (i.e., Thursday, Friday, and Saturday) was completed in order to report weekly variations on weekdays and the weekend. As confirmed in the literature, a 72 h food record can be used to assess nutrient intake because the record collects data for the typical or average diet [20]. Trained investigators filled in the 72 h food record in a face-to-face interview, during which individuals were asked to recall the food ingested in the preceding 72 h, including nutritional supplements and beverages. During the interviews, standard household measures and pictorial food models were employed to define amounts. Nutritional information was analyzed with Diet Source^®^ version 3.0, a nutritional computer application.

Using the method in Fung et al. [21], the resulting DASH score was based on food items emphasized or minimized in the DASH diet. The focus was on the following eight components: high intake of fruits, vegetables, nuts and legumes, whole grains, and low-fat dairy products and low intake of sodium, sweets, and red or processed meats [22]. The participants were classified based on the energy-adjusted quintile categories of their ingestion of these eight components. For sodium, sweets, and red or processed meats, scores of 5, 4, 3, 2, and 1 were assigned to those in the first (lowest), second, third, fourth, and fifth (highest) quintiles, respectively. In contrast, for fruits, vegetables, nuts and legumes, low-fat dairy products, and whole grains, the opposite scoring system was applied. The eight component scores were then added up to yield the total DASH score for each subject, which ranged from 8 to 40. A higher overall DASH score corresponds to greater adherence to the DASH dietary pattern [21,23].

### 2.5. Anthropometric Measurements and Physical Activity

Anthropometric parameters, including height, weight, body mass index (BMI), hip circumference, waist-to-hip ratio (WHR), and waist circumference (WC), were assessed according to the guidelines of the International Society for the Advancement of Kinanthropometry [24]. The weight of the subjects was measured with a self-calibrating Seca^®^ 861 class (III) digital floor scale, with a precision of up to 100 g. Their height was measured with a Seca^®^ 214 portable stadiometer. Participants were asked to stand in an upright position with their back and heels against the stadiometer and their head oriented on the Frankfurt plane. After the horizontal headpiece was placed on the top of their head, the BMI was calculated by dividing their weight by the square of their height (kg/m^2^).

The WC was measured at the horizontal plane midway between the lowest rib and the upper border of the iliac crest at the end of normal inspiration/expiration. Hip circumference was measured at the maximum width of the buttocks as viewed from the right side. A Seca^®^ automatic roll-up measuring tape with an accuracy of 1 mm was used for the WC and hip circumference measurements while the subjects remained in a standing position with their arms hanging at their sides at rest. WC had a CV% of 13.06%.

The WHR was calculated as their WC divided by their hip circumference. A body composition analyzer (TANITA Model BC-418 MA^®^, Tokyo, Japan) was used to estimate fat mass and visceral fat rating. This was done by measuring the bioimpedance of all participants. The CV% of % fat mass and visceral fat rating was of 37.87% and 86.25%, respectively. Visceral fat rating ranged from a minimum of 1 to a maximum of 59. A score of 1–12 indicates a healthy level of visceral fat, whereas a score of 13–59 indicates an excessive level of visceral fat [25]. The measurements were performed by the same trained researcher.

Physical activity was assessed by means of the Physical Activity Questionnaire for Older Children (PAQ-C), a seven-day recall questionnaire with high validity and moderate reliability [26]. The questionnaire consists of nine items, and each item is scored on a 5-point scale. A value from 1 to 5 was obtained for each of the nine items used in the physical activity composite score, and the mean of these nine items was the final PAQ activity score. Scores of 1, 2–4, and 5 indicated a low, moderate, and high physical activity, respectively.

### 2.6. Other Variables

The other variables studied were the presence or absence of parental obesity, which was determined by asking all participants to submit a medical certificate with this information. A variable related to religion was also included in our study, and each student self-identified the religion that he/she practiced (Islam or Christianity). This variable was measured by the Religious Attitude Questionnaire (*Cuestionario de Actitudes Religiosas*), developed and validated by Elzo [27].

### 2.7. Statistical Analysis

The participants were classified in three groups based on DASH score tertiles (*n* = 73, *n* = 102, and *n* = 69 in tertiles 1, 2, and 3, respectively). Continuous and categorical variables were compared in the tertiles by means of a one-way analysis of variance and the chi-squared test, respectively. In the two models, the multivariable-adjusted means of BP in DASH score tertiles were compared by performing an analysis of covariance (ANCOVA). In model 1, we adjusted the effects of sex and age as potential confounders, and in model 2, we controlled the effects of socioeconomic status (SES), parental obesity, PAQ-C summary score, WC, BMI Z-score, and energy intake. Pairwise differences in the mean BPs between the highest (T3) and lowest tertiles (T1) of the DASH score were examined by the Bonferroni post hoc test to adjust for multiple comparisons. All of the analyses were performed with version 24 of the SPSS software package (IBM, Armonk, NY, USA). A two-sided *p*-value <0.05 was considered statistically significant.

## 3. Results

The characteristics of the participants in the DASH score tertiles are listed in Table 1. Significant differences in religion, visceral fat rating, WC, and systolic BP were found in the tertiles (all *p* < 0.05). The mean systolic BP and the mean diastolic BP were 115.7 and 67.7, respectively, which yielded an overall mean BP of 91.7 mmHg. Coefficients of variation of % fat mass, WC, systolic BP, and diastolic BP were low, indicating that arithmetic mean is representative of the data set and this data is homogeneous, while visceral vat rating presented a high dispersion.

Table 2 shows the dietary intake of the participants by tertile of DASH score. Except for omega-3 and omega-6 fatty acids, significant differences were observed in all dietary variables measured in the DASH score tertiles (all *p* < 0.05). Those participants with lower adherence (T1) to the DASH dietary pattern showed a higher intake of total fat, saturated fatty acid (SFA), cholesterol, and sodium. In contrast, subjects with a stricter degree of adherence (T3) showed a dietary pattern characterized by a higher intake of potassium, magnesium, and calcium accompanied by a higher ingestion of fiber, fruits, vegetables, legumes, nuts, low-fat dairy products, and whole grains.

Table 3 lists the multivariable-adjusted means of the BP, visceral fat rating, and WC by DASH score tertile. In model 1, the multivariate analysis, adjusted for parental obesity, physical activity, and energy intake, showed that the mean systolic BP was lower as adherence to the DASH dietary pattern increased. This trend was also observed in the variables of visceral fat rating and WC. In model 2, adjusted for the confounding factors in model 1 as well as for sex and religion, the data reflected a very similar trend, though slightly better estimates and significance levels were found for systolic BP (*p* = 0.005) and WC (*p* = 0.003) with increasing adherence to the DASH dietary pattern among the university students. 

## 4. Discussion

To the best of our knowledge, this is the first study that evaluates the direct relationship between the DASH diet and variables such as BP, visceral fat rating, and WC in a sample population of Spanish university students. As reflected in our results, a stricter adherence to the DASH dietary pattern was associated with lower BP, visceral fat rating, and WC values. These results agree with Chiavaroli et al. [28], whose systematic review and meta-analysis found that, in controlled trials, the DASH diet was directly linked to a reduction in systolic BP (mean difference −5.2 mmHg (95% confidence interval (CI) −7.0 to −3.4)) and diastolic BP (−2.60 mmHg (−3.50 to −1.70)) as well as to an improved lipid profile, total cholesterol (−0.20 mmol/L (−0.31 to −0.10)), and low-density lipoprotein (LDL) cholesterol (−0.10 mmol/L (−0.20 to −0.01)).

Our findings are also consistent with those of previous studies of young people, in which stricter adherence to the DASH diet was associated with a lower incidence of metabolic syndrome (MetS) and its components, such as elevated systolic BP and a predominantly abdominal and visceral fat distribution [29]. This association between the DASH diet and BP levels can be explained by the low sodium content of the DASH diet, which decreases BP in this group [30]. Correspondingly, the meta-analysis performed by He et al. [31] demonstrated that dietary sodium intake is a modifiable risk factor for hypertension at any stage of life. Furthermore, lower sodium intake may lead to improved BP levels through different mechanisms [32].

In another study of type 2 diabetic adults who followed the DASH diet for eight weeks, greater adherence to this pattern led to a significant reduction in body weight, WC, systolic BP, and diastolic BP [33]. These results suggest that the DASH diet may be a powerful tool to prevent the development of cardiovascular risk factors, such as high BP and troncular obesity. Another interesting finding of this study was that the students that strictly followed the DASH dietary pattern ingested more potassium, magnesium, fiber, and calcium, accompanied by a higher consumption of fruits, vegetables, legumes, nuts, low-fat dairy products, and whole grains. Quite possibly, this dietary pattern characterized by an abundant intake of fruits and vegetables, especially leafy vegetables, helps to reduce BP levels through nonenzymatic generation of nitric oxide by inorganic nitrate [34].

Likewise, according to Streppel et al. [35], the high contents of potassium and magnesium, one of the characteristics of the DASH diet, may explain the diet’s beneficial effects on metabolism in general and on the lipid profile. In addition, according to Penton et al. [36], a high intake of potassium and magnesium may have antihypertensive effects derived from the ability of both minerals to induce vasodilation, reduce the release of renin at the kidney level, and establish a negative balance with sodium.

On the other hand, according to Bucher et al. [37], a high intake of calcium in subjects with high adherence to the DASH diet may act as a modulating factor of BP levels, specifically decreasing systolic BP, although this speculation remains controversial. In their review of randomized clinical trials, Cormick et al. [38] indicated that abundant calcium intake slightly reduced systolic BP and diastolic BP, especially in normotensive young adults. On the other hand, a higher intake of fruits, vegetables, and legumes, together with less SFA and more monounsaturated fatty acid consumption, may explain the beneficial effects of the DASH diet on the parameters of abdominal fat and visceral fat accumulation.

In addition, according to Hall [39], a high intake of mainly SFAs and nonesterified fatty acids may activate proinflammatory pathways, increasing oxidative stress and thus favoring endothelial dysfunction in such subjects. Based on these results, the DASH dietary pattern most likely exerts its beneficial effects through a combination of all these dietary factors. The fruits, vegetables, and other food items emphasized by the DASH diet contain numerous flavonoids and antioxidants, which can help to significantly reduce biomarkers of oxidative stress and inflammation [40,41], improve endothelial function, and thereby decrease BP levels [42]. However, the mechanisms through which the DASH diet acts on metabolic health are not fully understood and should be investigated in greater depth.

One limitation of this study is that its cross-sectional design does not allow an inference of causality between compliance with the DASH dietary pattern and BP. The small sample size may act as a mitigating factor in the detection of a possible association between adherence to the DASH dietary pattern and diastolic BP. However, this study has remarkable strengths, such as the use of standardized and validated instruments and methodological procedures and an appropriate sampling method.

## 5. Conclusions

Stricter adherence to the DASH dietary pattern was found to be associated with lower BP, visceral fat rating, and WC values in a sample population of young university students. These results suggest that the DASH dietary pattern may be a useful tool in daily clinical practice to prevent and identify cardiovascular risk factors, such as high BP and predominantly troncular obesity. As mechanisms through which the DASH diet acts on metabolic health are not as yet fully understood, prospective studies should be carried out to further confirm these findings.

## Figures and Tables

**Table 1:** Characteristics of the study participants in the tertiles of the DASH score.

Characteristics	All (*n* = 244)	DASH Score Tertiles			*p*-Value
		T1 (Lowest) (*n* = 73)	T2 (*n* = 102)	T3 (Highest) (*n* = 69)	
Age (years)	21.6 ± 2.85	21.6 ± 3.26	21.6 ± 2.69	21.6 ± 2.67	0.996
Sex					0.414
Male	85 (34.8)	21 (28.8)	39 (38.2)	25 (36.2)	
Female	159 (65.2)	52 (71.2)	63 (61.8)	44 (63.8)	
Religion					<0.001
Christian	131 (53.7)	47 (64.4)	61 (59.8)	23 (33.3)	
Muslim	113 (46.3)	26 (35.6)	41 (40.2)	46 (66.7)	
Parental obesity					0.185
Yes	52 (21.3)	17 (23.3)	21 (20.6)	14 (20.3)	
No	192 (78.7)	56 (76.7)	81 (79.4)	55 (79.7)	
PAQ-C summary score	4.0 ± 0.73	3.9 ± 0.79	4.0 ± 0.66	4.1 ± 0.75	0.126
BMI (kg/m^2^)	23.2 ± 3.62	23.1 ± 4.05	23.3 ± 3.14	23.1 ± 3.88	0.932
Fat mass (%)	23.1 ± 8.75	24.7 ± 8.46	23.1 ± 9.05	21.6 ± 8.68	0.138
Visceral fat rating	2.4 ± 2.07	2.6 ± 1.90	2.4 ± 1.81	2.3 ± 2.56	0.045
WC (cm)	77.7 ± 10.15	80.1 ± 9.15	77.6 ± 10.50	73.7 ± 10.53	0.023
Hip circumference (cm)	101.72 ± 10.44	103.65 ± 12.41	101.28 ± 9.63	100.23 ± 10.11	0.467
WHR	0.8 ± 0.09	0.9 ± 0.09	0.8 ± 0.09	0.7 ± 0.07	0.235
Systolic BP (mmHg)	115.7 ± 11.61	118.2 ± 13.33	114.4 ± 10.65	111.6 ± 10.12	0.010
Diastolic BP (mmHg)	67.7 ± 9.41	69.3 ± 12.13	66.7 ± 10.11	65.2 ± 9.60	0.549
Mean BP (mmHg)	91.7 ± 9.77	95.7 ± 11.23	93.2 ± 10.37	91.9 ± 9.17	0.295

Data are presented as *n* (%) or the mean ± standard deviation. The chi-squared test and one-way variance analysis were used to compare categorical and continuous variables between DASH score tertiles, respectively. DASH, Dietary Approaches to Stop Hypertension; PAQ-C, Physical Activity Questionnaire for Older Children; BMI, body mass index; WC, waist circumference; WHR, waist-to-hip ratio; BP, blood pressure.

**Table 2:** Dietary intake of the participants across tertiles of the DASH score.

Characteristics	All (*n* = 244)	DASH Score Tertiles			*p*-Value
		T1 (lowest) (*n* = 73)	T2 (*n* = 102)	T3 (highest) (*n* = 69)	
Energy (kcal/day)	2.0 ± 0.65	2.2 ± 0.56	2.0 ± 0.60	1.9 ± 0.80	0.011
**Nutrients (Daily Intake/1000 kcal)**					
Carbohydrate (g)	236.3 ± 86.82	236.8 ± 72.49	232.6 ± 78.25	241.1 ± 110.61	0.004
Fiber (g)	13.3 ± 5.91	12.4 ± 5.14	13.1 ± 5.53	14.6 ± 6.98	0.031
Protein (g)	84.3 ± 29.20	86.7 ± 23.46	84.7 ± 28.14	81.2 ± 31.65	0.030
Total fat (g)	84.1 ± 30.82	92.8 ± 31.33	82.4 ± 27.92	77.6 ± 32.69	0.010
SFA (g)	25.8 ± 10.95	29.4 ± 11.74	25.2 ± 9.75	22.9 ± 10.88	0.001
Omega-3 fatty acid (g)	0.6 ± 0.40	0.6 ± 0.35	0.6 ± 0.41	0.5 ± 0.43	0.357
Omega-6 fatty acid (g)	5.4 ± 3.10	5.6 ± 2.59	5.4 ± 3.26	5.2 ± 3.38	0.232
MFA (g)	30.4 ± 12.13	33.5 ± 13.18	30.7 ± 11.42	26.8 ± 11.19	0.005
Cholesterol (mg)	413.0 ± 182.65	452.0 ± 193.73	411.9 ± 161.64	373.5 ± 193.52	0.037
Calcium (mg)	833.5 ± 328.09	819.4 ± 407.14	832.2 ± 320.40	848.99 ± 249.79	0.002
Magnesium (mg)	231.9 ± 84.71	222.1 ± 65.85	229.4 ± 73.22	244.2± 113.24	<0.001
Potassium (g)	2.5 ± 0.93	2.4 ± 0.85	2.5 ± 0.82	2.6 ± 1.14	0.012
Sodium (g)	2.6 ± 1.2	2.8 ± 0.94	2.7 ± 0.89	2.4 ± 1.13	0.001
**Food Groups (Daily Intake/1000 kcal)**					
Sweets (g)	137.3 ± 145.89	155.2 ± 92.14	139.3 ± 89.72	117.6 ± 76.14	<0.001
Red or processed meats (g)	43.6 ± 25.97	49.3 ± 23.59	46.7 ± 25.85	34.8 ± 26.49	0.001
Fruits (g)	361.1 ± 221.75	263.7 ± 124.24	316.2 ± 195.64	503.6 ± 262.53	<0.001
Vegetables (g)	223.3 ± 124.10	167.1 ± 100.08	233.3 ± 109.30	269.6 ± 144.82	0.002
Nuts and legumes (g)	80.1 ± 56.91	60.9 ± 40.18	78.9 ± 61.51	100.7 ± 58.47	0.005
Low-fat dairy products (g)	167.2 ± 108.71	147.95 ± 80.98	151.5 ± 102.31	202.2 ± 133.24	<0.001
Whole grains (g)	32.1 ± 22.76	24.4 ± 16.07	31.6 ± 24.61	40.3 ± 23.39	0.005

Data appear as the mean ± standard deviation. A one-way variance analysis was used to compare all dietary variables in different DASH score tertiles. DASH, Dietary Approaches to Stop Hypertension; SFA, saturated fatty acid; MFA, monounsaturated fatty acid.

**Table 3:** Multivariable-adjusted means of blood pressure, visceral fat rating, and waist circumference by tertile of the DASH score (*n* = 244).

	DASH Score Tertiles			*p*-Value	Pairwise Difference [T3–T1]	*p*-Value
	T1 (Lowest)	T2	T3 (Highest)			
**Model 1**						
Systolic BP (mmHg)	116.2 (113.3; 119.2)	114.6 (111.9; 117.6)	111.5 (108.8; 114.2)	0.009	−4.70 (−8.8; −0.6)	0.010
Diastolic BP (mmHg)	67.7 (65.2; 70.2)	65.5 (62.8; 68.2)	63.1 (60.3; 65.9)	0.236	−4.60 (−8.2; −1.0)	0.345
Visceral fat rating	2.5 (2.1; 2.9)	2.3 (1.9; 2.7)	2.1 (1.6; 2.6)	0.027	−0.4 (−3.3; 2.6)	0.041
WC (cm)	78.9 (75.8; 81.9)	77.2 (74.4; 79.9)	76.2 (73.3; 79.1)	0.008	−2.7 (−6.9; 1.5)	0.035
**Model 2**						
Systolic BP (mmHg)	115.8 (113.3; 118.4)	113.2 (110.7; 115.7)	111.4 (108.9; 113.8)	0.005	−4.36 (−7.3; −1.4)	0.004
Diastolic BP (mmHg)	67.1 (65.9; 68.3)	65.2 (62.8; 67.6)	63.0 (61.7; 65.3)	0.245	−4.1 (−7.4; −0.8)	0.365
Visceral fat rating	2.5 (2.1; 2.9)	2.3 (2.0; 2.7)	2.1 (1.7; 2.5)	0.025	−0.4 (−3.5; 2.7)	0.024
WC (cm)	78.7 (75.8; 81.6)	77.4 (73.5; 81.3)	75.5 (71.8; 79.2)	0.003	−3.2 (−7.20; 0.7)	0.019

Data are presented as the mean (95% confidence interval). Multivariable-adjusted means of BP, visceral fat rating, and WC were compared between DASH score tertiles using analysis of covariance (ANCOVA) models. Model 1 was adjusted for parental obesity, the physical activity questionnaire summary score, and energy intake. Model 2 was adjusted for the confounders in model 1 as well as for sex and religion. Pairwise differences in the means of BP, visceral fat rating, and WC between the upper (T3) and lower third (T1) of the DASH score were analyzed with the Bonferroni post hoc test. DASH, Dietary Approaches to Stop Hypertension; BP, blood pressure; WC, waist circumference.
